# Comparative analysis of electromagnetic navigation bronchoscopy versus computed tomography‐guided lung puncture for the sampling of indeterminate pulmonary nodules in the middle of an anatomic lung segment: A cohort study

**DOI:** 10.1111/1759-7714.14726

**Published:** 2022-12-04

**Authors:** Shuliang Zhang, Feilong Guo, Hongjin Wang, Maohui Chen, Guanglei Huang, Yong Zhu, Wei Zheng, Bin Zheng, Chun Chen

**Affiliations:** ^1^ Fujian Key Laboratory of Cardiothoracic Surgery (Fujian Medical University), Fujian Province University Fuzhou China; ^2^ Department of Thoracic Surgery Fujian Medical University Union Hospital Fuzhou China

**Keywords:** anatomic lung segment, computed tomography‐guided lung puncture, electromagnetic navigation bronchoscopy, pneumothorax

## Abstract

**Background:**

To compare the diagnostic positive rate and complication rate between the electromagnetic navigation bronchoscopy (ENB) technique and computed tomography (CT)‐guided lung puncture for the biopsy of lung nodules located in the middle of an anatomic lung segment.

**Methods:**

Electronic medical records of 114 patients who underwent lung nodule biopsy between June 2021 and June 2022 were retrospectively evaluated. In all patients, the nodules were located in the middle third lung segment. To compare the diagnostic positive and complication rates between the two biopsy modalities performed in this lung region, clinical data, complication rates, nodule pathology, and imaging results were reviewed based on nodule characteristics retrieved from the electronic medical records.

**Results:**

Ninety‐three patients underwent CT‐guided lung puncture, while the remaining 21 patients underwent the ENB technique. No significant difference was observed in the diagnostic positive rate between the two groups (73.6 and 76.1%, respectively). In the CT‐guided lung puncture group, pneumothorax incidence, tube placement, postoperative hemorrhage, and symptomatic hemorrhage rates were 16.1, 6.5, 6.5, and 1.1%, respectively. In contrast, no complications occurred in the ENB group.

**Conclusions:**

The ENB technique is a safe and effective method for performing biopsies of pulmonary nodules with a diagnostic positive rate comparable to that of CT‐guided lung puncture and with a lower postoperative complication rate.

## Graphical abstract

Computed tomography (CT)‐guided lung puncture or electromagnetic navigation bronchoscopy (ENB) can be used to sample indeterminate pulmonary nodules in the middle of an anatomic lung segment. ENB is safe and feasible for sampling nodules in the middle of an anatomic segment. ENB has a high diagnostic positive rate with a low risk of complications.

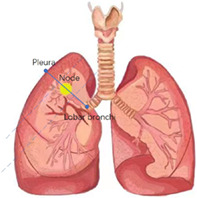

## INTRODUCTION

The widespread use of thin‐slice computed tomography (CT) for diagnosis has led to a substantial increase in the detection rate of pulmonary nodules.^1^, ^2^ There is a high possibility that most pulmonary nodules detected on imaging are clinical manifestations of lung cancer, thus requiring further definitive diagnosis to guide subsequent treatment. Therefore, the pathological biopsy of pulmonary nodules is crucial. For operable patients, those with benign nodules can be spared to undergo surgical trauma, while biopsy results could be used to guide the development of neoadjuvant or translational treatment protocols for those who are potentially operable or have locally advanced cancer. For late inoperable patients, pulmonary nodule biopsy serves as an important theoretical basis for subsequent treatment.

There is, however, no consensus on the technique used for performing pathological biopsies of pulmonary nodules, and thoracic surgeons choose different biopsy methods based on a combination of various factors, including imaging findings, postoperative complications, technical difficulty, and cost. The commonly used biopsy technique for peripheral pulmonary nodules is CT‐guided lung puncture, which has a high sensitivity of 81%–97% for diagnosing malignancy.^3^ However, this procedure frequently leads to severe complications such as pneumothorax and bleeding. In a retrospective study of 15 865 individuals by Wiener et al., pneumothorax incidence, tube placement rate, and postoperative hemothorax incidence after CT‐guided lung puncture were 15, 6, and 1%, respectively; however, the probability of bleeding was low, and 17.8% of patients with bleeding required blood transfusion.^1^ Furthermore, nodules with a smaller diameter and deeper location make the operation more difficult, and the possibility of postoperative complications increases significantly.^4^, ^5^, ^6^ In contrast to CT‐guided lung puncture, electromagnetic navigation bronchoscopy (ENB) is a relatively novel pathological biopsy method with greater safety and feasibility, as confirmed by several studies. In a meta‐analysis of 39 studies by Wang Memoli et al., the positive rate of ENB for the diagnosis of pulmonary nodules was 70%, while the incidence of pneumothorax was only 1.5% (in contrast to 15% in CT‐guided lung puncture), thus demonstrating that it is a safer and more feasible new biopsy technique.^7^

Previous comparative studies, however, did not consider the interference caused by nodule location on the positive rate of the biopsy method. None of the current studies that compared ENB with CT‐guided lung needle biopsy considered the effect of nodule location on biopsy results. To date, only a few studies have focused on the comparative study of nodule distance from the pleura, but no consensus was reached among the studies on this aspect of the relationship between the positive diagnosis rate and the nodule distance from the pleura. Therefore, in the present study, we performed a comparative analysis between ENB and CT‐guided lung biopsy methods for sampling nodules located in the middle of an anatomic lung segment; the study aimed to suggest a suitable biopsy technique to provide a better comprehensive understanding of the nature of lung nodules, which could enable physicians to select appropriate clinical diagnosis and treatment strategies for patients.

## METHODS

### Ethical statement

Because of the retrospective nature of our study, the Institutional Review Board of Fujian Medical University Union Hospital waived the need to obtain informed consent from the patients for accessing their medical records.

### Lung nodule zonation

The bronchial origin of the lobe in which the nodule was located was used as the starting point, passing through the center of the nodule and finally reaching the visceral pleura as an endpoint. This distance was then divided into three equal parts for classifying the nodule location: inner, middle, and outer (Figure 2).

### Patient cohort and data collection

**FIGURE 1:**
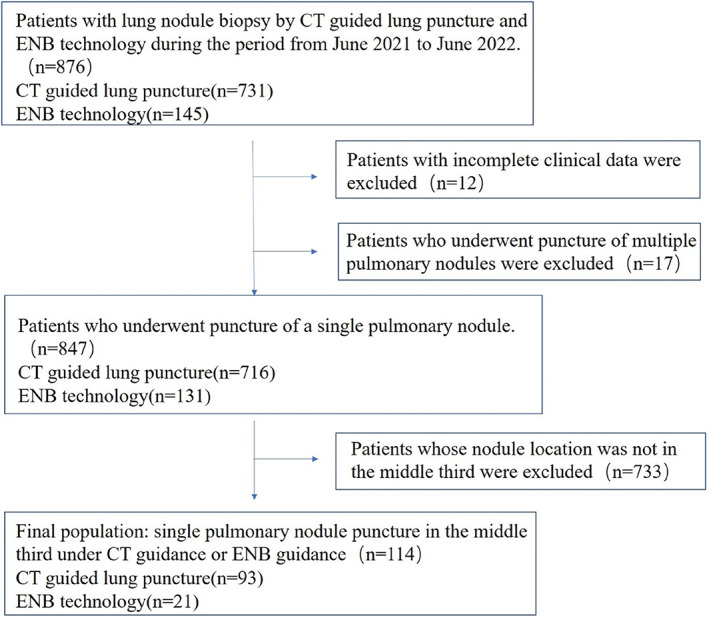
Flow chart of study profile

Patients who presented to the Fujian Medical University Union Hospital between June 2021 and June 2022 with pulmonary CT findings suggestive of pulmonary nodules with a maximum nodule diameter of ≥8 mm were included in this study, and their clinical data were retrieved from electronic medical records. The flow chart is shown in Figure 1. The patients were grouped according to their biopsy method (CT‐guided lung puncture or ENB). The collected demographic data included the following: age; gender; body mass index (BMI); American Society of Anesthesiologists (ASA) score; medical history of hypertension and diabetes mellitus; history of thoracic surgery; history of smoking and alcohol consumption (past or current smokers/drinkers and never smokers/drinkers); prebiopsy evaluation data, including nodule size, nodule density (ground‐glass, partly solid, and solid), and nodule shape (both lobulation and spiculation, lobulation alone, spiculation alone); nodule location; type of pathology after biopsy; and occurrence of postoperative pneumothorax and bleeding .

**FIGURE 2:**
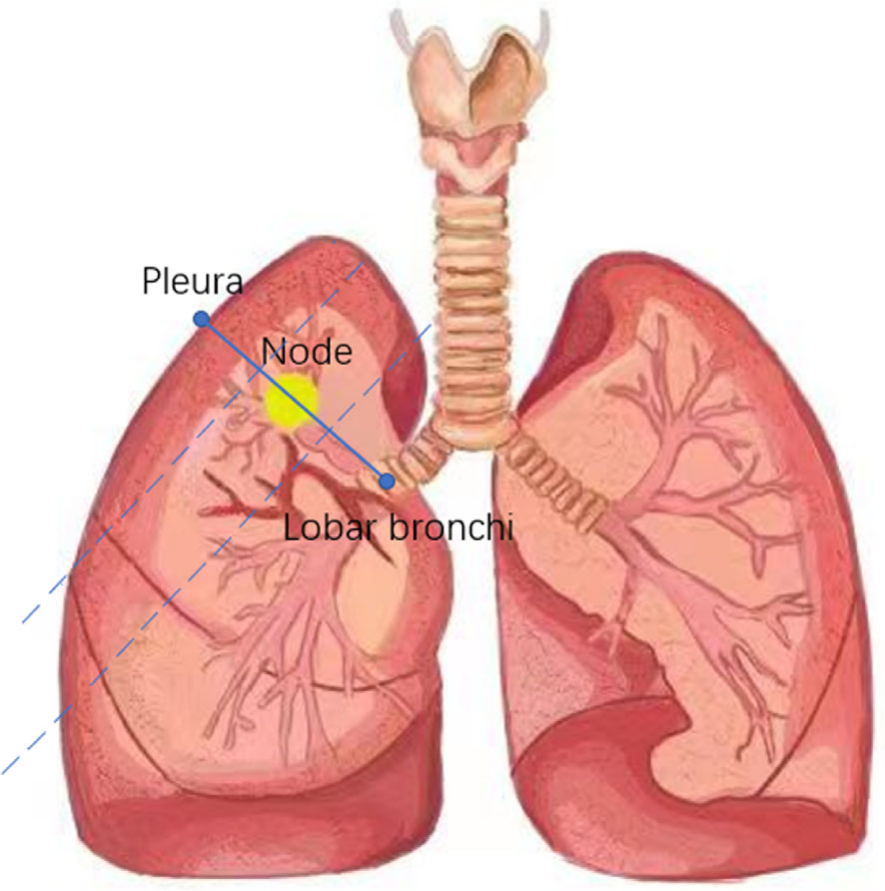
The bronchial origin of the lobe in which the nodule was located was used as the starting point, passing through the center of the nodule and finally reaching the visceral pleura as an endpoint. This distance was then equally divided into three parts: inner, middle and outer

### Biopsy methods

#### CT‐guided lung puncture

Depending on the location and accessibility of the lesion, the patient was placed in a suitable position (prone, supine, or lateral) on the CT machine. Preoperatively, a CT scan in the intended area was performed to locate the needle entry point. After inducing local anesthesia with lidocaine, the needle was left in place, and a CT scan was performed to confirm the orientation and angle of the needle and to calculate the needle depth. The needle was removed after satisfactory placement of the trocar until it reached the appropriate depth. A CT scan was again performed to assess the position of the trocar relative to the tumor location. The trocar was then advanced into the tumor tissue and removed after performing a biopsy to obtain sufficient tissue specimen. The CT scan was performed again postoperatively, and the patient was monitored for the occurrence of pneumothorax or hemothorax  .

**FIGURE 3:**
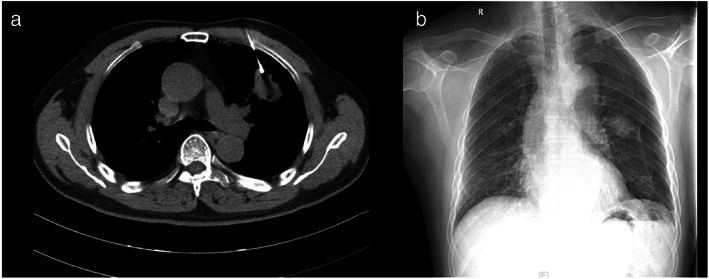
Depicts the case of a patient with severe pneumothorax requiring tube drainage after computed tomography (CT)‐guided lung puncture. A 64‐year‐old patient with lung adenocarcinoma was found to have a nodule in the left upper lobe on CT imaging; the patient presented with 40% lung collapse after lung puncture, and his symptoms were relieved after closed chest drainage

#### ENB

The procedure was performed as follows:

1. The CT imaging data were transferred to the medical imaging workstation, and the CT data were imported in the DICOM format into the electromagnetic navigation planning software, which is based on bronchial, vascular, and pleural segmentation and reconstruction technology, to achieve preoperative three‐dimensional (3D) imaging.
2. 3D reconstruction was performed to generate virtual bronchial tree images. The target lesion morphology was outlined, and the location of the target lesion was marked. The target bronchus leading to the target lesion was prioritized, and the ENB navigation path was accordingly set.
3. The guidewire with the positioning information was inserted into the bronchoscope working channel such that the lead tip slightly extended out of the working channel. The guidewire was placed just above the carina and walked separately on the left and right bronchi for registration of the electromagnetic position information. After the registration process was completed, the bronchoscope was again placed in the carina, and electromagnetic navigation was performed after three respiratory curves while viewing the fit of the endoscopic real‐time image with the virtual endoscopic image.
4. Intraoperatively, the bronchoscope was advanced as far as possible to the peripheral bronchus according to the path guided in real‐time by electromagnetic navigation. When no further advancement was possible, the guidewire was controlled for its direction of travel by adjusting the bronchoscope helix and/or the rotating ring, advancing stepwise, deeper into each level of the bronchus, toward the location of the target lesion.
5. When the ENB system indicated that the front end of the guidewire had reached the target lesion, the ultrasound probe was removed, and the positions of the biopsy forceps and the cell brush were marked. The biopsy forceps and the cell brush were then advanced to the target lesion through the positioning catheter extension tube for biopsy and brush examination; the cell smear was performed immediately. The above procedure was repeated until satisfactory pathological samples were obtained, and the process was completed. The bronchial lumen was observed, and the operation was considered to have been completed when no active bleeding was noted.

### Statistical analysis

The clinical parameters evaluated in this study included the biopsy sensitivity and the complication rate, which were defined as the percentage of positive patients after biopsy and the number of patients with complications, respectively. The study data were analyzed using SPSS software (SPSS 18.0). Categorical variables were expressed as percentages and analyzed by the χ^2^ test. Continuous variables were expressed as mean ± standard deviation (SD) and analyzed by *t*‐test. A *p*‐value of ≤0.05 was considered statistically significant .

**FIGURE 4:**
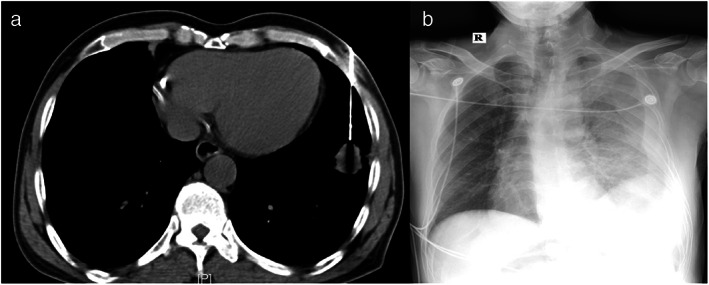
Depicts the case of a patient with severe hemothorax after computed tomography (CT)‐guided lung puncture. A 56‐year‐old patient was found to have a nodule in the left lower lobe identified on CT imaging, and the nodule was sampled by lung puncture. Because of the longer surgical path, the biopsy procedure was more complex and lengthier, leading to the development of progressive hemothorax

## RESULTS

From June 2021 to June 2022, 876 patients underwent CT‐guided lung puncture and ENB in the Fujian Medical University Union Hospital, including 114 patients with nodules located in the middle third lung segment. Of these 114 patients, 93 patients underwent CT‐guided lung puncture and 21 patients underwent ENB for obtaining biopsy specimens. The demographic data of the study subjects and the imaging characteristics of the nodules are presented in Table 1. The positive rates in the CT‐guided lung puncture and ENB groups were 73.1% (68/93) and 76.1% (16/21), respectively. In the CT‐guided lung puncture group, 61 patients were diagnosed to have malignant tumors, seven patients had benign nodules, and 25 patients showed negative results. In the ENB group, 15 patients were diagnosed to have malignant nodules, one patient had a benign nodule, and five patients showed negative results. Adenocarcinoma accounted for the highest proportion of malignant tumors in the CT‐guided lung puncture and ENB groups (73.8% and 86.6%, respectively). Table 2 presents the classification and proportion of pathological findings after biopsy. Table 3 shows the effect of nodule characteristics on the diagnostic positive rate of both biopsy methods, and no significant correlation was observed between both biopsy methods and nodule characteristics. Table 4 presents the complication rates after biopsy in both groups. In the CT‐guided lung puncture group, pneumothorax occurred in 15 patients (16.1%); tube placement was required in six patients (6.5%); postoperative bleeding occurred in six patients (6.5%) (Figure 3); and severe hemothorax developed in one patient (1.1%), who was admitted to the Intensive Care Unit and underwent emergency surgery (Figure 4). None of the patients in the ENB group developed pneumothorax or hemorrhage. No other complications were detected in this study.

**TABLE 1:** Patient characteristics

Patient characteristic	CT‐guided group *n* = 93	ENB‐guided group *n* = 21	*p*‐value
Age, years	62.18 ± 11.33	60.76 ± 10.67	0.601
Sex			0.711
Male	49 (52.6%)	12 (57.1%)	
Female	44 (47.4%)	9 (42.9%)	
BMI^a^, kg/m^2^	23.23 ± 3.67	22.68 ± 2.42	0.518
ASA score^a^			0.905
1	62 (66.7%)	15 (71.4%)	
2	25 (26.9%)	5 (23.8%)	
3	6 (6.4%)	1 (4.8%)	
Distance from pleura, mm	37.60 ± 8.12	36.62 ± 5.78	0.603
Lobe location			0.852
Right upper	28 (30.1%)	4 (19.0%)	
Right middle	7 (7.5%)	2 (9.5%)	
Right lower	23 (24.7%)	6 (28.5%)	
Left upper	19 (20.4%)	4 (19.0%)	
Left lower	16 (17.3%)	5 (24.0%)	
Nodule diameter^a^, cm			0.947
<1	16 (17.2%)	4 (19.0%)	
1 ≤ diameter < 2	49 (52.7%)	11 (52.4%)	
2 ≤ diameter < 3	21(22.6%)	5 (23.8%)	
3 ≤ diameter < 4	4 (4.3%)	1 (4.8%)	
≥4	3 (3.2%)	NA	
Nodule density^a^			0.472
Ground glass	2 (2.1%)	1 (4.7%)	
Part solid	12 (12.9%)	1 (4.7%)	
Solid	79 (85.0%)	19 (90.6%)	
Nodule shape^a^			0.320
Lobulation and spiculation	23 (24.7%)	6 (28.5%)	
Lobulation	23 (24.7%)	3 (14.3%)	
Spiculation	9 (9.6%)	5 (23.8%)	
Irregular	20 (21.5%)	5 (23.8%)	
Circularity	18 (19.5%)	2 (9.6%)	
Diabetes			0.645
Yes	10 (10.8%)	3 (14.3%)	
No	83 (89.2%)	18 (85.7%)	
Hypertension			0.352
Yes	27 (29.1%)	4 (19.1%)	
No	66 (70.9%)	17 (80.9%)	
Smoking			0.903
Yes	21 (22.6%)	5 (23.8%)	
No	72 (77.4%)	16 (76.2%)	
Drinking			0.488
Yes	34 (36.5%)	6 (28.5%)	
No	59 (63.5%)	15 (71.4%)	

^a^ ASA score, American Society of Anesthesiologists score; BMI, body mass index; Nodule diameter, refers to the largest nodule size.

**TABLE 2:** Nodule diagnoses and comparison between sampling methods

Parameter	CT‐guided group	ENB‐guided group	*p*‐value
Diagnosis			0.772
Malignant	61 (65.6%)	15 (71.4%)	
Benign	7 (7.6%)	1 (4.8%)	
Unknown	25 (26.8%)	5 (23.8%)	
Type of malignant diagnosis			0.483
Adenocarcinoma	45 (73.8%)	13 (86.6%)	
Squamous cell carcinoma	4 (6.5%)	1 (6.7%)	
Other^a^	12 (19.7%)	1 (6.7%)	
Type of benign diagnosis			0.537
Tuberculosis infection	2 (28.6%)	0 (0%)	
Fungal infection/granuloma	5 (71.4%)	1 (100%)	

^a^ Other malignant diagnoses included metastases, lymphoma, small cell carcinoma, large cell carcinoma, mucoepidermoid carcinoma, leiomyosarcoma and mucinous adenocarcinoma.

**TABLE 3:** Impact of nodule characteristics on diagnostic yield of biopsy

	CT‐guided group		ENB‐guided group	
Diagnostic yield	Patients (%)	*p*‐value	Patients (%)	*p*‐value
Distance from pleura^a^, mm		0.154		0.132
<30 mm	92.9 (13/14)		100 (2/2)	
30–40 mm	66.7 (30/45)		61.5 (8/13)	
>40 mm	73.5 (25/34)		100 (6/6)	
Lobe location		0.107		0.822
Right upper	67.8 (19/28)		75 (3/4)	
Right middle	42.8 (3/7)		100 (2/2)	
Right lower	69.5 (16/23)		83.3 (5/6)	
Left upper	78.9 (15/19)		75 (3/4)	
Left lower	93.7 (15/16)		60 (3/5)	
Nodule diameter^a^, cm		0.664		0.934
<1	75.0 (12/16)		75.0 (3/4)	
1 ≤ diameter<2	71.4 (35/49)		72.7 (8/11)	
2 ≤ diameter<3	76.1 (16/21)		80 (4/5)	
3 ≤ diameter<4	50 (2/4)		100 (1/1)	
≥4	100 (3/3)		NA	
Nodule density		0.638		0.707
Ground‐glass	50 (1/2)		73.6 (14/19)	
Part solid	66.6 (8/12)		100 (1/1)	
Solid	74.6 (59/79)		100 (1/1)	
Nodule shape		0.739		0.177
Lobulation and spiculation	69.5 (16/23)		100 (5/5)	
Lobulation	82.6 (19/23)		66.6 (2/3)	
Spiculation	77.7 (7/9)		40 (2/5)	
Irregular	65 (13/20)		80 (4/5)	
Circularity	72.2 (13/18)		100 (2/2)	

*Note*: Data in parentheses are numerator/denominator. NA, not applicable.

^a^ Nodule diameter, refers to the largest nodule size.

**TABLE 4:** Complications as a function of biopsy method

Parameter	CT‐guided group (%)	ENB‐guided group (%)	*p*‐value
Any pneumothorax	16.1 (15)	0	0.168
Pneumothorax requiring chest tube/admission	6.5 (6)	0	
Any hemorrhage	6.5 (6)	0	0.361
Symptomatic hemorrhage	1.1 (1)	0	

*Note*: Data in parentheses are number of patients.

## DISCUSSION

ENB is a novel biopsy technique that has gradually emerged in recent years for the biopsy of pulmonary nodules through image‐guided flexible catheters and dedicated navigation software systems.^8^ Among the diagnostic methods for the biopsy of peripheral lung nodules, CT‐guided lung puncture is the most preferred technique because of its high accuracy. Previous studies reported a diagnostic rate of 67% to 97% and a pooled diagnostic rate of 92% for CT‐guided needle biopsy of pulmonary nodules, whereas the diagnostic rate of ENB ranged from 46% to 86%, and the pooled diagnostic rate was only 70%.^7^, ^9^ However, a recent study refuted previous findings, suggesting that the diagnostic success rate of ENB can reach 89% and that this technique is not inferior to CT‐guided lung puncture.^10^ In clinical practice, CT‐guided lung puncture was found to have regional limitations: cases in which the nodule is partially abutting on the top of the pleura, for which the puncture path should pass through the first intercostal space, have a significantly higher probability of developing a severe complication; moreover, in the scapular area, CT‐guided lung puncture cannot be performed by conventional manipulation, which greatly increases the complexity of the surgery. The ENB passes through the natural channels of the bronchus and can theoretically reach any location within the lung, thus having few blind areas for biopsy.

Many previous studies have reported the results of comparison between CT‐guided lung puncture and ENB. However, these studies did not specifically demarcate the location where the nodule was located, thus indicating some nodule selection bias, which is a relevant factor to be considered for the wide fluctuation of positive rates. A retrospective study of 295 patients by Bhatt et al. reported a contrast in the diagnostic positive rate of CT‐guided lung puncture and ENB (86% versus 66%), wherein the diagnostic positive rate of CT‐guided lung biopsy was much higher than that of ENB. Bhatt et al. suggested an interesting conclusion: the distance between the nodule and pleura was a critical factor that affected the diagnostic positive rate of CT‐guided lung biopsy technique, and the diagnostic positive rate increased with the increase in the distance; in contrast, the diagnostic positive rate of the ENB group did not show a similar correlation.^11^ Previous studies have also suggested that the distance of the nodule from the pleura is one of the factors influencing the diagnostic positive rate.^12^ However, we found that the distance from the pleura tended to be the shortest distance between the measured nodule and the visceral pleura, with a deviation from the path taken at the time of puncture, which might be a reason for its use as a critical factor. Therefore, our study adopted a new approach of lung nodule partition: using the bronchial opening of the lobe in which the nodule is located as the starting point, passing through the center of the nodule, and finally reaching the visceral pleura as an endpoint. This distance was equally divided into three parts: inner, middle, and outer. Compared to the length of the distance between the nodule and the pleura, this partitioning method can effectively avoid the errors that occur, thus making the contrast results more credible. Based on our previous clinical experience, we found that lesions located in the inner third location have a high accuracy rate and a very low complication rate when detected with ENB, while CT‐guided biopsy requires a long distance to reach this area. This increases the uncertainty during the operation; moreover, the probability of damage to the blood vessel and bronchus is significantly higher, and the decreased accuracy rate is often accompanied by the occurrence of complications. Therefore, the present study findings suggest that ENB is more advantageous than CT‐guided lung puncture in this surgical area. In contrast, lesions in the outer third region, potentially due to the requirement for deeper and larger distances, are observed with a lower accuracy rate with ENB and an increased accuracy rate and a significantly lower complication rate with CT‐guided lung puncture. Therefore, CT‐guided lung puncture should be preferred in this region. However, because the middle third region is in a junctional position, it is inconclusive which biopsy modality should be recommended for this region.

Our results revealed that the diagnostic positive rate showed no significant difference between CT‐guided lung puncture and ENB for nodules located in the middle third segment (73.1% vs. 76.1%), although the diagnostic positive rate of ENB was slightly higher than that of CT‐guided lung puncture. Postoperative complications were significantly more common for CT‐guided lung puncture than for ENB. We believe that the selection of cases for CT‐guided lung puncture and ENB correlates with the outcomes: the deeper nodule location in the middle third region and the difficulty encountered by CT‐guided lung puncture in precise localization lead to an increase in negative results for diagnosis; in contrast, the ENB is more precise in its diagnosis as the puncture is performed only at the nodule site according to transbronchial walking under navigation. We also found that the diagnostic positive rate of the two biopsy methods did not show a lobar tendency, whereas previous studies reported that nodules located in the upper lobe of both lungs and the right middle lobe would have a higher diagnostic positive rate with ENB, while CT‐guided lung puncture did not show this tendency.^11^, ^13^, ^14^ This difference is probably because of the small number of cases in our study.

Pneumothorax with hemorrhage is a common complication after lung nodule biopsy.^15^, ^16^, ^17^ In our study, pneumothorax was observed in 15 patients (16.1%) who underwent CT‐guided lung puncture, including six patients (6.5%) who required tube placement for drainage. This finding is similar to the results of a meta‐analysis reported by Dibardino et al., where 20.5% (range: 4–62%) of patients presented with pneumothorax, and the rate of tube placement was 7.3% (range: 0–31%). In the present study, patients who underwent biopsy with the ENB technique did not present with pneumothorax; Wiener et al. also reported that the incidence of pneumothorax and tube placement was 1.5% and 0.6%, respectively, for patients who underwent biopsy with the ENB technique.^7^ Although the occurrence of pneumothorax is related to underlying lung diseases such as emphysema and chronic obstructive pulmonary disease,^18^, ^19^ the incidence of pneumothorax in the ENB group remained significantly lower than that in the CT‐guided lung puncture after adjustment for population baseline in the study of Bhatt et al. Regarding post biopsy hemorrhage, the mean symptomatic hemorrhage rate for CT‐guided lung puncture was 2.8%,^9^ whereas this rate might be much higher for asymptomatic hemorrhage cases not detected after biopsy.^7^

The present study has some limitations: our data were derived from patients who presented to a single large tertiary hospital, indicating that the results may not be broadly generalizable. Our sample size was small, and the findings, although statistically significant, did not allow us to infer clinically meaningful conclusions. This was a retrospective clinical study, and therefore, the findings are required to be validated through future prospective studies. However, we consider that this research is the first step toward future studies on the comparison between ENB and CT‐guided lung puncture to help in clinical decision‐making for effective patient treatment.

In conclusion, to the best of our knowledge, the present study is the first to compare the diagnostic rates and postoperative complications of CT‐guided lung puncture and ENB for the biopsy of nodules located in the middle third segment of the lung. The diagnostic rate of ENB is slightly higher than that of CT‐guided lung puncture for lesions located in the middle third segment of the lung, and the ENB has high safety and a low complication rate. Based on the findings of the present study, we believe that the ENB technique should be considered more frequently and preferentially used for the diagnosis of lesions in the middle of an anatomic lung segment.

## AUTHOR CONTRIBUTION

Contributions of each author: (I) Conception and design: S Zhang, F Guo, H Wang; (II) Administrative support: S Zhang, F Guo, H Wang, B Zheng, C Chen; (III) Provision of study materials or patients: S Zhang, F Guo, H Wang; (IV) Collection and assembly of data: S Zhang, F Guo, H Wang, G Huang, W Zheng, B Zheng, C Chen; (V) Data analysis and interpretation: All authors; (VI) Manuscript writing: All authors; (VII) Final approval of manuscript: All authors. S Zhang and C Chen accept direct responsibility for the manuscript. S Zhang, F Guo, H Wang contributed equally to this work, and should be considered as co‐first authors.

## CONFLICT OF INTEREST

The authors declare there are no conflicts of interest.
